# The association between bilirubin and hypertension among a Chinese ageing cohort: a prospective follow-up study

**DOI:** 10.1186/s12967-022-03309-7

**Published:** 2022-03-04

**Authors:** Chen Tang, Hanxiang Jiang, Bin Zhao, Yi Lin, Shengnan Lin, Tianmu Chen, Yanhua Su, Yiqin Zhang, Lina Zhou, Lianmeng Li, Jincheng Lin, Zhonghua Lu, Yao Wang, Zeyu Zhao, Longjian Liu, Yuxin Wang, Jie Zhang, Heqing Shen

**Affiliations:** 1 State Key Laboratory of Molecular Vaccinology and Molecular Diagnotics, School of Public Health, Xiamen University, No. 4221-117 South Xiang’an Road, Xiang’an District, Xiamen, 361102 Fujian People’s Republic of China; 2 Xiamen Guankou Hospital, No. 270 Jingshan Road, Jimei District, Xiamen, 361023 Fujian People’s Republic of China; 3 Clinical Medical Laboratory, Xiang’an Hospital of Xiamen University, No. 2000, East Xiang’an Road, Xiamen, 361102 Fujian People’s Republic of China; 4 Department of Nephrology, The Second Affiliated Hospital of Xiamen Medical College, No. 566 Shengguang Road, Jimei District, Xiamen, 361021 Fujian People’s Republic of China; 5 Department of Epidemiology and Biostatistics, Dornsife School of Public Health, Drexel University, 3141 Chestnut Street, Philadelphia, PA USA

**Keywords:** Ageing, Bilirubin, Cohort study, Hypertension

## Abstract

**Background:**

Hypertension is highly prevalent and associated with the elevated risks of cardiovascular diseases, dementia, and physical disabilities among adults. Although the correlation between bilirubin and hypertension has been reported, the observation in quinquagenarian population is scarce. We aimed to examine bilirubin-hypertension association in Guankou Ageing Cohort Study.

**Methods:**

Participants ≥ 55 years were recruited and their questionnaires and physical examination data were collected. Kaplan–Meier survival analysis and Cox proportional hazards regression were implemented to assess the hypertension risk. The non-liner dose–response relationships of bilirubin-hypertension were determined by restricted cubic spline (RCS) models. Receiver operating characteristic (ROC) curves and multiple factors analysis (MFA) were performed to evaluate the predictive abilities.

**Results:**

1881 eligible participants (male 43.75%, female 56.25%) with the median age of 61.00 (59.00–66.00) were included. The hazard ratio (HR, 95% *CI*) of serum total bilirubin (STB) and unconjugated bilirubin (UCB) were 1.03 (1.01–1.05) and 1.05 (1.03–1.07), while conjugated bilirubin (CB) showed a weak protective effect with the HR of 0.96 (0.92–0.99), and the associations remained significant in all models. RCS analyses further indicated the similar bidirectional effects of STB and UCB with the cut-off of 12.17 μmol/L and 8.59 μmol/L, while CB exhibited inverse bidirectional dose–response relationship with a cut-off of 3.47 μmol/L. ROC curves and MFA showed baseline STB combined with age, BMI, and waist circumference could well discriminate the low and high of hypertension risk.

**Conclusions:**

Our findings suggested the higher levels of total and unconjugated bilirubin were hazardous factors of hypertension, while an inverse effect presented when more bilirubin was conjugated.

**Supplementary Information:**

The online version contains supplementary material available at 10.1186/s12967-022-03309-7.

## Background

Ageing is defined as a gradual decline in the ability to maintain whole-body homeostasis, causing the onset of ageing-related diseases (ARDs) and eventually death [1]. As the ageing tendency of the population accelerates, the elderlies are becoming an increasingly important subpopulation that merits special attention regarding health and social issues [2]. Hypertension, a representative ageing syndrome which is common in the middle old-ages (quinquagenarian), is characterized by persistently elevated systemic arterial blood pressure (BP) and may be accompanied by functional or organic damage to the heart, brain, and kidney, which is ranked third as a cause of disability-adjusted life-years and affected over 1/5 adults (26.4%) worldwide [3–5]. Although the age-standardized prevalence of hypertension decreased in high-income countries over the past decade, it has been increasing in low- and middle-income countries [5]. In China, along with the urbanization, economic growth, and the population ageing, the prevalence of hypertension has been elevated markedly. Some surveys have revealed that 26.6–33.6% of the total population was diagnosed with hypertension [6, 7], which estimated to cause 23 million deaths per year (increased 89% compared with 1990) [8]. Besides the high prevalence, the rates of awareness, treatment, and control were also inadequate, which exerts heavy burdens on the public health system in China, even in the entire world [9]. In view of the severe situations, discovering novel hypertension-related biomarkers and distinguishing high-/low-risk population through biomarker-based models would better facilitate the prevention and control of hypertension [10].

Bilirubin (C_33_H_36_N_4_O_6_), conventionally considered as the ultimate product of heme metabolism, is produced starting from the breakdown of red blood cells to generate carbon monoxide, iron, and biliverdin. Biliverdin is an unstable intermediate and rapidly converts into bilirubin by biliverdin reductases [11]. In hepatocytes, bilirubin is conjugated with glucuronic acid by uridine-diphosphate glucuronosyltransferase 1A1 (UGT1A1), known as conjugated bilirubin (CB) [12]. Comparing with unconjugated bilirubin (UCB), CB is soluble, which can be filtered and excreted via kidney but does not cross the brain barrier [13]. Animal experiments showed bilirubin mediated cytoprotection effects, including anti-oxidation and anti-inflammatory in vivo [14]. Several epidemiological studies have also shown the inverse correlation between bilirubin and the risk of cardiovascular diseases [15, 16]. However, bilirubin presents the potential cytotoxic effects either. Toxicological studies manifested that high serum concentration of bilirubin would bound to and deposited on various tissues, and further pleaded to deleterious events such as jaundice, mental disorders, cerebral palsy, brain damage, and even death [17]. Beyond that, hyperbilirubinemia was associated with a worse world health organization functional class, higher right atrial pressure, higher brain natriuretic peptide, and a larger Doppler right ventricular index [18]. Hence, the subtle role of this substance in human metabolism keeps unclear.

Until now, some epidemiological studies have aimed to uncover the relationship between bilirubin and BP among children and middle-age adults. However, very limited studies have been conducted among the ageing population, especially in China [19–21]. Given the potential indication of bilirubin towards hypertension, we aimed to examine the association between the baseline levels of bilirubin and the incident risk of hypertension with the adjustments for key covariates on the dataset of the Guankou Ageing Cohort Study (GACS).

## Material and methods

### Study population

The research protocol of the GACS has been approved by the Medical Ethics Committee of School of Medicine, Xiamen University. All procedures were conducted in accordance with the Declaration of Helsinki, and all patients were required to provide written informed consent prior to participation. No adverse events were reported during or after completion of the study. The GACS was a prospective dynamic cohort study nested in the public health service system in Xiamen. Through tracing the disease process of ARDs (hypertension, diabetes mellitus, and dementia), this cohort study aimed at recognizing risk factors in the ageing process and providing clues to possible pathogenesis. We recruited the ageing inhabitants (≥ 55 years) from a rural area of Jimei District in Xiamen, China. The participants have relatively stable sociological features and can be followed over a long period. Subjects underwent annual comprehensive health check-ups in the Xiamen Guankou hospital from July 1st, 2013, until onset of hypertension, death, or the end of observation (December 31st, 2019). The participants’ lifestyle questionnaires, demographic information, and comprehensive medical check-up data were collected along with the biological samples. The data obtained at the initial medical check-up were served as the baseline information.

### Data collection

Before recruitment, all investigators and staff members accepted specific trainings to be familiar with the aims of the study as well as the application methods of equipment. The standardized questionnaire developed by the School of Public Health, Xiamen University, was used to determine the information regarding of marriage status, lifestyle factors (e.g., smoking, drinking and physical exercise). All questionnaires were collected through computer-assisted face-to-face interviews during clinic visits under the guidance of investigators. After at least 10-min relaxation, three consecutive BP readings were obtained with a five-minute interval and the average was used as BP value. Subjects with BP variation beyond 10 mm Hg among the measurements were required to take a repeated measurement in three days later. Suspected hypertensive patients were asked to take further consultation until confirmation. Waist circumference (WC), which is the horizontal circumference of the mid-point line between the lowest rib and the upper edge of the iliac crest (about 1 cm to the upper edge of navel), was measured with errors of 0.5 cm. Body height and weight were measured when the subjects were taken in light clothing and without shoes (with errors 0.5 cm and 0.1 kg, respectively). Body mass index (BMI) was computed as weight in kg divided by height in m^2^. A 5-mL of venous blood was drawn from each subject was drawn into sodium citrate anticoagulant tube after overnight fasting (about 12 h) and then sent immediately to biochemistry laboratory in an ice cooler for the further processing and analyses.

### Definitions and participant classifications

Hypertension was defined as sustainably systolic blood pressure (SBP) ≥ 140 mm Hg and/or diastolic blood pressure (DBP) ≥ 90 mm Hg taken in clinic or with a history of taking antihypertensive medications. Cases of incident hypertension were defined as those who had no baseline hypertension and were diagnosed during follow-up. Prehypertension was defined as SBP 120 to 139 mm Hg and DBP 80 to 89 mm Hg without antihypertensive medication. Stage 1 hypertension was defined as SBP 140 to 159 mm Hg and DBP 90 to 99 mm Hg, Stage 2 as 160 to 179 mm Hg and 100 to 109 mm Hg, and Stage 3 as ≥ 180 mm Hg and ≥ 110 mm Hg [22]. Four subsets of participants in GACS were initially categorized based on the STB, CB, and UCB quartiles at baseline to examine the overall relationship between hypertension and bilirubin. Type 2 diabetes mellitus (T2DM) was defined as fasting plasma glucose at least 7.0 mmol/L or if the subjects being on antidiabetic agents currently [23]. And hyperuricemia was diagnosed as serum uric acid ≥ 420 μmol/L (7.0 mg/dL) in males and serum uric acid ≥ 360 μmol/L (6.0 mg/dL) in females [24].

### Statistical analysis

Multiple imputation by chained equations (MICE) was implemented to fill out the missing covariate values prior to statistical analysis [25]. Kolmogorov–Smirnov test and Levene’s test were performed to assess the normality and homogeneity of variance, respectively. All data are displayed as mean standard deviation (± SD), median (lower and upper quartiles), and frequencies (percentages) depending on the type of data. Student’s t-test or one-way analysis of variance (ANOVA), and Manne-Whitney U test or Kruskal–Wallis test, and Pearson’s Chi-square test were performed for comparing normally distributed continuous variables, uneven distributed variables, and categorical variables, respectively. The incidence density of hypertension was calculated as the total event number divided by the sum of follow-ups (per 100 person-years).

The Kaplan–Meier log-rank analyses and Cox proportional hazards regression models were applied after adjusting for potential key confounders to calculate the hazard rations (HR) and 95% confidence intervals (*CI*) for studying the relationship between hypertensive incidence and bilirubin levels. As the levels of fasting plasma glucose (FPG), STB, CB, and serum creatinine (SCr) in the population did not meet normal distribution, different types of data transformation methods were performed before the above-mentioned analyses. These data were converted by base 10 logarithmic, Box-Cox [26], and square root (SQRT) transformation to obtain normal distributions. Among them, the calculation formula of Box-Cox transformation is shown as follows:

$$y (w, \lambda ) =\left\{\begin{array}{c}\frac{{w}^{\lambda }-1}{\lambda }, \lambda \ne 0,\\ \mathrm{ln}w, \lambda =0.\end{array}\right.$$

where *y* represents the novel variable obtained after Box-Cox transformation, *w* is the original continuous dependent variable, and *λ* is the transformation parameter to be identified [27].

A multivariable Cox model with restricted cubic spine (RCS) with 4 knots were further constructed to check the bilirubin-hypertension dose–response associations to avoid the inappropriate linearity assumptions [28], as the RCS model is a smoothly joined sum of polynomial functions that do not assume linearity of the relationship. The threshold was determined as the identification of the risk function inflexion point. The 95% *CI* was derived by bootstrap resampling. The calculation formula of multivariable Cox proportional hazards regression is described below [29]:

$$ h\left( {t,X} \right) \, = h_{0} \left( t \right){\text{ exp }}(\beta_{{1}} X_{{1}} + \beta_{{2}} X_{{2}} + \cdots + \beta_{{\text{m}}} X_{{\text{m}}} ) $$

The above formula gave the underlying hazard at time *t* for subject *i* with covariates (explanatory variables) *X*_i_.

To evaluate if the model of bilirubin combined with other statistically significant risk factors could improve sensitivity to distinguish the high and low risk of hypertensive group, we calculated the area under the ROC curve (AUC) for each model by using pROC package [30]. The goodness of model fits was evaluated by the ANOVA and Akaike information criterion (AIC). And the distinguishing ability of hypertensive or non-hypertensive population was further determined by subsequent unsupervised clustering analysis based on multiple factor analysis (MFA) algorithm [31]. Two-tailed probability values < 0.05 were considered being statistically significant at 0.05 level. We performed all analyses using R software version 4.0.5 (R foundation, Vienna, Austria) for Windows and SPSS software version 26.0 (IBM SPSS Inc., Chicago, IL, USA).

### Patient and public involvement

Neither patient was involved in setting the research question or the outcome measures, nor of them have involved in developing the plans for recruitment, design, or implementation of the study. No patient was asked of advice on interpreting or writing up of the results.

## Results

### Baseline characteristics of participants

**Fig. 1:**
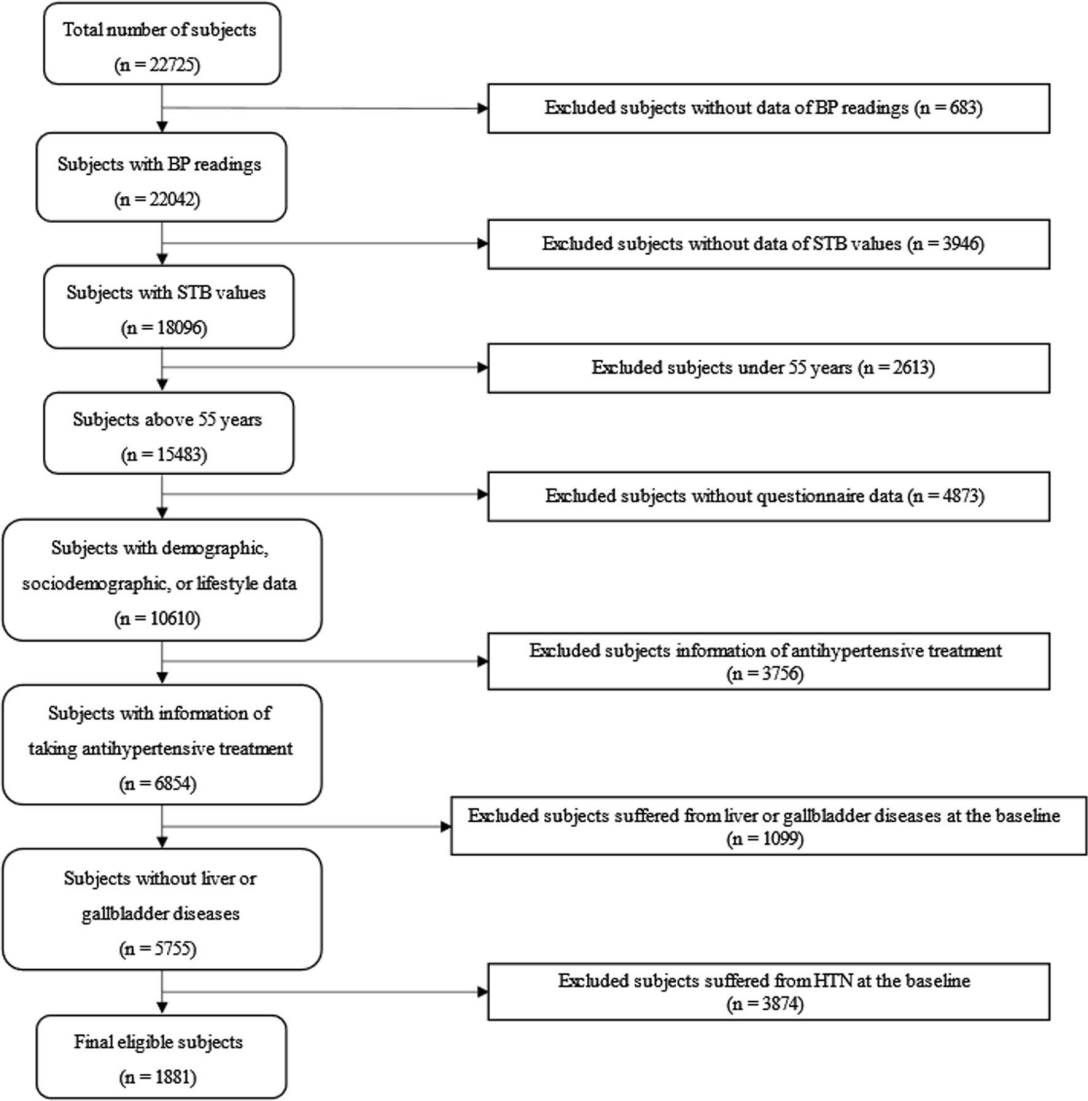
Flow diagram of participant inclusion and exclusion criteria in the GACS. BP, blood pressure; HTN, hypertension; STB, serum total bilirubin

At the beginning of this study, 22,725 subjects were included in the GACS, who took part in at least 2 times of the follow-up visits and medical tests. As shown in Fig. 1, we excluded 20,844 subjects according to the following criteria: (1) lack of BP readings (n = 683); (2) lack of STB (n = 3946); (3) under the age of 55 years (n = 2613); (4) lack of demographic, sociodemographic, or lifestyle data (n = 4873); (5) lack of information on whether taking antihypertensive treatment (n = 3756); (6) suffered from liver diseases (hepatitis, alcoholic liver, cirrhosis) or gallbladder diseases (cholecystitis, gallstones, choledocholithiasis) which resulted in the elevation of bilirubin levels (n = 1099) during the follow-up process. As displayed in Additional file 1: Table S1, the hypertensive prevalence at the baseline was 67.32% (41.79% in males; 58.21% in females) and there was no gender composition difference between the 2 subgroups (χ^2^ = 2.00, *P* = 0.158). We further excluded 3874 subjects suffered from hypertension at the baseline, thus, 1881 subjects were enrolled in the next stage statistical analyses.

Missing values of BMI (n = 12), WC (n = 43), leukocyte (n = 64), platelet (n = 63), hemoglobin (n = 60), FPG (n = 12), alanine transaminase (ALT) (n = 3), aspartate aminotransferase (AST) (n = 3), and alcohol consumption status (n = 10) were interpolated using MICE method. The median age of subjects at recruitment was 61.00 years, with 823 (43.75%; 62.00 years) males. Here, smoking status was classified as “current smoker” (smoked at least one cigarette per day for over 12 months), “former smoker” (former daily smoker who quit smoking > 6 months), and “non-smoker” (including occasional smoking and former occasional smoking); status of alcohol consumption was categorized as “current drinker” (individuals who drunk at least one cup (approximately 125 mL) of alcoholic beverages (beer, wine, and liquors) per day for over 12 months) and “never drinker” (including occasional drinking and former occasional drinking). The frequency of exercise was roughly defined as how many times they took various forms of physical exercise per week (≥ once monthly as the low, ≥ once weekly as the medium, and daily as the high). The study population included 491 current smokers (26.10%), 311 current drinkers (16.62%), and 779 participants (41.41%) with the medium or high frequency of exercise. Table 1 showed the specific baseline information of the subjects in the GACS, which revealed that male individuals had higher age, DBP, leukocyte, hemoglobin, ALT, STB, CB, and SCr. Female participants exhibited larger percentages of widowhood, non-smoker, non-drinker, as well as low and medium exercise frequency compared with male participants. The baseline SBP of male (125.00 (117.00–132.00)) and female participants (124.00 (116.75–131.00)) were not statistically different (*P* = 0.289), while the DBP of male subjects (76.00 (71.00–81.00)) were slightly higher (*P* < 0.001) than females (75.00 (70.00–80.00)). In addition, the prevalence of T2DM and hyperuricemia were not significantly different between genders (*P*_T2DM_ = 0.750; *P*_hyperuricemia_ = 0.629).

**Table 1:** Characteristics of the 1881eligible participants at recruitment

Variables	Overall	Male	Female	*P* value
	N = 1881	n = 823 (43.75%)	n = 1058 (56.25%)	
Age (years)	61.00 (59.00–66.00)	62.00 (60.00–67.00)	60.00 (59.00–65.00)	< 0.001
WC (cm)	81.00 (75.00–88.00)	81.00 (75.00–87.00)	81.00 (75.00–87.00)	0.482
BMI (kg/m^2^)	24.13 ± 60.92	21.97 (20.06–23.99)	22.52 (20.78–24.92)	< 0.001
SBP (mm Hg)	124.00 (117.00–131.00)	125.00 (117.00–132.00)	124.00 (116.75–131.00)	0.289
DBP (mm Hg)	76.00 (70.00–80.00)	76.00 (71.00–81.00)	75.00 (70.00–80.00)	< 0.001
Leukocyte (× 10^9^/L)	5.90 (5.10–7.01)	6.40 (5.46–7.50)	5.60 (4.80–6.52)	< 0.001
Platelet (× 10^9^/L)	217.00 (190.00–252.00)	213.00 (186.00–246.00)	220.00 (192.75–255.00)	0.001
Hemoglobin (g/L)	135.00 (126.00–144.00)	143.00 (135.00–151.00)	130.00 (123.00–136.00)	< 0.001
FPG (mmol/L)	4.99 (4.50–5.60)	4.85 (4.40–5.47)	5.10 (4.50–5.70)	< 0.001
ALT (U/L)	20.90 (16.30–27.50)	21.90 (17.00–28.60)	20.40 (15.70–26.33)	< 0.001
AST (U/L)	21.00 (18.00–24.10)	21.00 (18.00–24.10)	21.00 (18.00–24.20)	0.650
Albumin (g/L)	44.50 (42.90–46.00)	44.50 (42.90–46.00)	44.60 (42.88–46.10)	0.386
STB (μmol/L)	11.90 (9.25–15.20)	12.50 (9.50–16.00)	11.50 (9.00–14.50)	< 0.001
CB (μmol/L)	3.20 (2.20–4.50)	3.60 (2.50–5.00)	2.90 (1.90–3.90)	< 0.001
UCB (μmol/L)	8.30 (5.90–11.50)	8.40 (5.90–11.60)	8.20 (5.90–11.10)	0.316
SCr (μmol/L)	62.80 (51.70–75.80)	72.50 (64.00–83.80)	54.70 (47.30–64.43)	< 0.001
TC (mmol/L)	5.00 (4.20–6.00)	4.84 (4.19–5.55)	5.16 (4.53–5.82)	< 0.001
TG (mmol/L)	5.03 (4.40–5.71)	0.99 (0.74–1.43)	1.08 (0.79–1.52)	0.001
T2DM (n, %)	(176) 9.36	79 (9.60)	97 (9.17)	0.750
Hyperuricemia (n, %)	468 (24.88)	200 (24.30)	268 (25.35)	0.629
Marriage Status (n, %)				< 0.001
Married	1638 (87.10)	767 (93.20)	871 (82.33)	
Widowed	229 (12.20)	49 (5.95)	180 (17.01)	
Unspecified	14 (0.70)	7 (0.85)	7 (0.66)	
Smoking Status (n, %)				< 0.001
Non-smoker	1337 (71.08)	294 (35.72)	1043 (98.58)	
Former Smoker	53 (2.82)	52 (6.32)	1 (0.09)	
Current Smoker	491 (26.10)	477 (57.96)	14 (1.32)	
Drink Status (n, %)				< 0.001
Non-drinker	1560 (83.38)	553 (67.69)	1007 (95.54)	
Current Drinker	311 (16.62)	264 (32.31)	47 (4.46)	
Exercise Frequency				< 0.001
Low	1102 (58.59)	488 (52.30)	614 (58.03)	
Medium	322 (17.12)	124 (15.07)	198 (18.71)	
High	457 (24.29)	211 (25.63)	246 (23.26)	

Normally distributed variables with even variance were presented as mean ± SD, skewed variables as median (lower quartile to upper quartile), and categorical variables as n (%). Continuous variables were compared using Student’s t-test or Mann–Whitney U test depending on distribution. Pearson’s χ^2^ tests were used to compare categorical values

HTN, hypertension; WC, waist circumference; BMI, body mass index; SBP, systolic blood pressure; DBP, diastolic blood pressure; FPG, fasting plasm blood; ALT, alanine aminotransferase; AST, aspartate aminotransferase; STB, serum total bilirubin; CB, conjugated bilirubin; UCB, unconjugated bilirubin; SCr, serum creatinine; TC, total cholesterol; TG, triglyceride; T2DM, type 2 diabetes mellitus

### Kaplan–Meier analysis

**Fig. 2:**
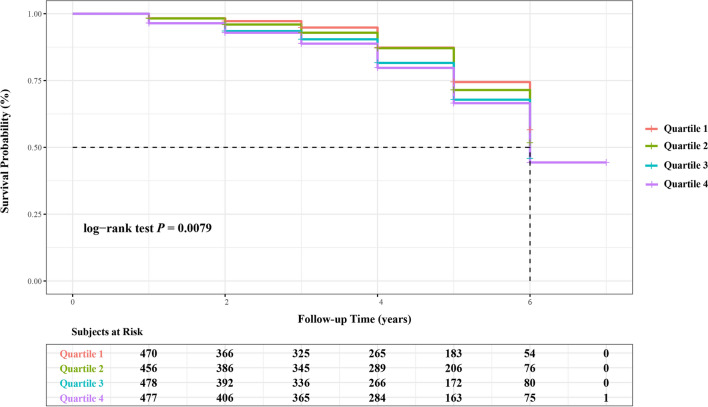
Cumulative risk curves for hypertension incidence by Kaplan–Meier analysis. Groups of STB concentration were defined as: Quartile 1, < 9.3 (μmol/L); Quartile 2, 9.3 ~ 11.8 (μmol/L); Quartile 3, 11.9 ~ 15.2 (μmol/L); Quartile 4, > 15.3 (μmol/L)

These 1881 participants were further divided into 4 subgroups on the basis of the STB quartiles, and the total and the average length of follow-up was 6916 and 3.68 ± 1.69 person-years, respectively. Their detail information and the hypertensive incidence were summarized in Table 2, which revealed the individuals who had the higher STB level were likely to have the higher values of hemoglobin, ALT, AST, CB, and UCB. When the STB level increased, the incident density of hypertension also presented an elevating trend (*P* = 0.018). During the seven-year of follow-up, 435 cases were diagnosed with hypertension and the overall accumulative incident rate was 6.29 per 100 person-years (5.97 and 6.51 per 100 person-years in men and women, respectively) in the GACS. The cumulative hazards of hypertensive incidence were generated by Kaplan–Meier log-rank analysis was shown in Fig. 2, and the results of pairwise comparisons between quartiles were shown in Additional file 1: Table S4. As expected, the cumulative hazards increased with follow-up time. And the positive correlation seemed to exist between STB levels and the hypertensive incidence in the GACS, as the highest STB quartile exhibited the highest onset risk for hypertension among the four subgroups. Moreover, the hypertension risk in quartile 1 was significantly lower than quartile 3 (*p* = 0.013) and quartile 4 (*p* = 0.003), while the similar pattern could also be observed between quartile 2 and quartile 4 (*p* = 0.028).

**Table 2:** Baseline characteristics of initial non-HTN participants according to baseline bilirubin quartiles

Variable	Overall	STB quartiles				
		1 (Lowest)	2	3	4	***P*** value
		< 9.3 (μmol/L)	9.3 ~ 11.8 (μmol/L)	11.9 ~ 15.2 (μmol/L)	> 15.2 (μmol/L)	
	N = 1881	n = 470	n = 456	n = 478	n = 477	
Age (years)	61.00 (59.00–66.00)	62.00 (59.00–67.00)	61.00 (59.25–66.00)	61.00 (59.00–66.00)	61.00 (59.00–65.00)	0.107
Length of follow-up (Person-years)	3.68 ± 1.69	3.54 ± 1.73	3.86 ± 1.67	3.61 ± 1.72	3.71 ± 1.61	0.025
Male (n, %)	823 (43.75)	189 (40.21)	174 (38.16)	212 (44.35)	248 (51.99)	< 0.001
WC (cm)	81.00 (75.00–88.00)	81.00 (74.00–87.00)	81.00 (76.00–88.00)	81.00 (75.00–87.00)	80.00 (74.00–87.00)	0.211
BMI (kg/m^2^)	22.26 (20.45–24.46)	22.15 (20.44–24.34)	22.66 (20.95–24.94)	22.21 (20.44–24.44)	22.04 (20.03–24.25)	0.007
SBP (mm Hg)	127.00 (117.00–138.00)	127.00 (116.75–138.00)	129.00 (118.00–139.00)	125.50 (116.00–137.00)	127.00 (117.50–137.50)	0.094
DBP (mm Hg)	78.00 (72.00–84.00)	77.00 (71.00–84.00)	78.00 (72.00–85.00)	78.00 (72.00–84.00)	78.00 (72.00–85.00)	0.389
Leukocyte (× 10^9^/L)	5.90 (5.10–7.01)	5.90 (5.29–7.10)	5.80 (5.00–7.10)	6.00 (5.20–7.10)	5.96 (4.90–7.90)	0.169
Platelet (× 10^9^/L)	217.00 (190.00–252.00)	225.00 (201.00–261.25)	217.00 (190.25–251.00)	215.00 (185.00–250.25)	212.00 (184.00–242.00)	< 0.001
Hemoglobin (g/L)	135.00 (126.00–144.00)	132.00 (124.75–142.00)	134.00 (126.00–142.00)	136.00 (126.75–145.00)	138.00 (129.50–147.00)	< 0.001
FPG (mmol/L)	4.99 (4.50–5.60)	4.94 (4.50–5.60)	4.90 (4.40–5.50)	4.99 (4.67–5.60)	5.10 (4.50–5.70)	0.278
ALT (U/L)	20.90 (16.30–27.50)	19.20 (14.68–25.63)	20.70 (16.40–27.10)	21.90 (17.00–29.25)	21.90 (17.65–27.50)	< 0.001
AST (U/L)	21.00 (18.00–24.10)	19.95 (17.30–24.00)	20.00 (17.93–23.30)	21.00 (18.20–25.00)	21.00 (19.00–25.00)	< 0.001
Albumin (g/L)	44.50 (42.90–46.00)	44.45 (42.50–45.93)	44.20 (42.60–46.00)	44.60 (42.90–46.10)	44.80 (43.30–46.10)	0.024
CB (μmol/L)	3.20 (2.20–4.50)	2.40 (1.60–3.10)	3.10 (2.30–3.70)	3.50 (2.48–4.50)	4.50 (2.85–5.80)	< 0.001
UCB (μmol/L)	8.50 (5.90–11.50)	5.00 (3.70–6.00)	7.40 (6.50–8.38)	9.90 (8.60–11.13)	14.00 (11.60–16.75)	< 0.001
SCr (μmol/L)	62.80 (51.70–75.80)	63.70 (51.48–77.33)	60.05 (48.10–73.75)	62.55 (52.43–75.43)	64.40 (54.00–77.00)	0.001
TC (mmol/L)	5.03 (4.40–5.71)	4.94 (4.20–5.65)	5.07 (4.35–5.70)	5.07 (4.43–5.72)	5.04 (4.46–5.76)	0.135
TG (mmol/L)	1.04 (0.77–1.48)	1.08 (0.81–1.62)	1.07 (0.78–1.46)	1.03 (0.77–1.49)	0.99 (0.72–1.39)	0.007
T2DM (n, %)	178 (9.46)	52 (11.06)	42 (9.21)	43 (8.81)	41 (8.78)	0.510
Hyperuricemia (n, %)	468 (24.88)	82 (17.44)	107 (23.46)	142 (29.71)	137 (28.78)	< 0.001
Marriage (n, %)						0.976
Married	1638 (87.08)	405 (86.17)	395 (86.62)	421 (88.08)	417 (87.42)	
Widowed	229 (12.17)	61 (12.98)	58 (12.72)	53 (11.09)	57 (11.95)	
Unspecified	14 (0.74)	4 (0.85)	3 (0.66)	4 (0.84)	3 (0.63)	
Smoking Status (n, %)						0.069
Non-smoker	1331 (70.76)	335 (71.28)	335 (73.46)	340 (71.13)	321 (67.30)	
Former Smoker	53 (2.82)	9 (1.91)	10 (2.19)	11 (2.30)	23 (4.82)	
Current Smoker	491 (26.10)	125 (26.60)	110 (24.12)	124 (25.94)	132 (27.67)	
Drinking Status (n, %)						0.019
Non-drinker	1560 (82.93)	396 (84.26)	388 (85.09)	402 (84.10)	374 (78.41)	
Current Drinker	311 (16.53)	70 (14.89)	67 (14.69)	73 (15.27)	101 (21.74)	
Exercise Frequency (n, %)						0.013
Low	1102 (58.59)	307 (65.32)	257 (56.36)	269 (56.28)	269 (56.39)	
Medium	322 (17.12)	55 (11.70)	87 (19.08)	89 (18.62)	91 (19.08)	
High	457 (24.30)	108 (22.98)	112 (24.56)	120 (25.10)	117 (24.53)	
Incident HTN (n, %)	435 (6.29)	81 (4.87)	104 (5.92)	121 (7.01)	129 (7.28)	0.018

Normally distributed variables are presented as mean ± SD, skewed variables as median (interquartile range), and categorical variables as n (%). Incidence rate was calculated as the number of hypertension incident cases divided by 100 person-years of follow-up. Continuous variables were compared using one-way ANOVA or Kruskal–Wallis test depending on distribution. Pearson’s χ^2^ tests were used to compare categorical values

HTN, hypertension; WC, waist circumference; BMI, body mass index; SBP, systolic blood pressure; DBP, diastolic blood pressure; FPG, fasting plasm blood; ALT, alanine aminotransferase; AST, aspartate aminotransferase; STB, serum total bilirubin; CB, conjugated bilirubin; UCB, unconjugated bilirubin; SCr, serum creatinine; TC, total cholesterol; TG, triglyceride; T2DM, type 2 diabetes mellitus

### Cox proportion hazards regression model

Cox proportional hazards regression model was further performed to elaborate the quantitative profile between bilirubin and the hypertension risks. Multicollinearity diagnostics was conducted to detect the multicollinearity between key variables. The values of Tolerance (TOL) and Variance Inflation Factor (VIF) greater than 0.1 and 10 indicates the multicollinearity is acceptable [32–34]. The variable of hyperuricemia was not included in the model construction due to its values of TOL and VIF exceeded thresholds. As summarized in Table 3, variables of BMI, WC, FPG, AST, STB, CB, UCB, SCr, and current smoking were significantly associated with the incident risk of hypertension, and current smoking (1.99 (1.60–2.46)) and DM (1.67 (1.28–2.19)) were the two most significant risk factors. HR (95% *CI*) for STB was 1.03 (1.01–1.05), while for BMI, WC, FPG, UCB, STB, AST, and SCr, these ratios were 1.23 (1.00–1.26), 1.08 (1.07–1.09), 1.07 (1.05–1.09), 1.05 (1.03–1.07), 1.03 (1.01–1.05), 1.02 (1.01–1.03), and 1.01 (1.00–1.01), respectively. However, CB showed the weak inverse relationship with incident hypertension with the HR of 0.96 (0.92–0.99).

**Table 3:** Hazard ratios of serum total bilirubin levels for hypertension in the GACS

Variables	HR (95% *CI*)	*P* value
Female	1.08 (0.89–1.31)	0.430
Age	1.01 (0.99–1.03)	0.244
BMI	1.23 (1.20–1.26)	< 0.001
WC	1.08 (1.07–1.09)	< 0.001
SBP	1.00 (0.99–1.00)	0.962
DBP	1.00 (0.99–1.01)	0.885
FPG	1.07 (1.05–1.09)	< 0.001
AST	1.02 (1.01–1.03)	0.003
STB	1.03 (1.01–1.05)	0.002
CB	0.96 (0.92–0.99)	0.022
UCB	1.05 (1.03–1.07)	< 0.001
SCr	1.01 (1.00–1.01)	0.005
T2DM	1.67 (1.28–2.19)	< 0.001
Current Smoker	1.99 (1.60–2.46)	< 0.001

BMI, body mass index; WC, waist circumference; SBP, systolic blood pressure; DBP, diastolic blood pressure; FPG, fasting plasm glucose; AST, aspartate aminotransferase; STB, serum total bilirubin; CB, conjugated bilirubin; UCB, unconjugated bilirubin; SCr, serum creatinine; T2DM, type 2 diabetes mellitus

^*^P < 0.05 was considered statistically significant

Table 4 showed the HRs calculated by multivariable adjusted Cox proportional hazards regressions. Raw data of FPG, STB, and SCr were transformed by base 10 logarithmic, Box-Cox (λ = − 0.418), and SQRT to meet the requirement of normal and homoscedastic distribution prior to analysis. Our findings suggested that the hypertensive risk for subjects in the highest STB quartile was approximately 1.5 times as that of the lowest quartile in the crude model (HR 1.49, 95% *CI* 1.13–1.97), and the correlation remained significant in all 4 models after the adjustment of multivariable. And after adjusting for age, gender, BMI, WC, baseline BP level (SBP and DBP), DM, FPG, AST, and SCr (Model 3), the HR of the highest quartile was 1.76 (95% *CI* 1.32–2.34). The similar positive relationship could also be observed between hypertensive risk and UCB levels; individuals in the highest UCB quartile group exhibited 2.26 times higher incident risk compared with those in the lowest level group in model 1 (Additional file 1: Table S3). However, as it was displayed in Additional file 1: Table S2, a weak negative association could be observed between the hypertension incident risks and serum CB levels with the gradual declined tendency of HRs, which hinted the risk of hypertension decreased with the increasement of CB levels.

**Table 4:** Prospective analysis of associations between STB levels and hypertension incidence

	STB quartiles				
	1 < 9.3 μmol/L	2 9.3 ~ 11.8 μmol/L	3 11.9 ~ 15.2 μmol/L	4 > 15.3 μmol/L	*P* for trend
Crude Model	Reference	1.14 (0.85–1.52)	1.40 (1.05–1.85)	1.49 (1.13–1.97)	0.017
Model 1	Reference	1.10 (0.82–1.47)	1.49 (1.12–1.97)	1.68 (1.27–2.22)	< 0.001
Model 2	Reference	1.11 (0.83–1.48)	1.50 (1.13–1.99)	1.68 (1.27–2.23)	< 0.001
Model 3	Reference	1.21 (0.90–1.63)	1.60 (1.20–2.12)	1.76 (1.32–2.34)	< 0.001
Model 4	Reference	1.19 (0.89–1.60)	1.46 (1.10–1.94)	1.68 (1.26–2.23)	0.002

Multivariable-adjusted Cox regression models were used to assess the hypertension incidence risk by STB quartiles. Crude model: Only included STB level at the baseline. Multivariable model 1: Included variables of age, gender, BMI, and WC on the basis of the Crude model. Multivariable model 2: Further included variables of SBP, DBP, and DM at baseline on the basis of the Multivariable model 1. Multivariable model 3: Further included variables of FPG_*log10*_, AST, and SCr_*SQRT*_ on the basis of the Multivariable model 2. Multivariable model 4: Further included the variable of smoking status on the basis of the Multivariable model 3

STB, serum total bilirubin; BMI, body mass index; WC, waist circumference; SBP, systolic blood pressure; DBP, diastolic blood pressure; T2DM, type 2 diabetes mellitus; AST, aspartate aminotransferase; FPG, fasting plasma glucose; SCr, serum creatinine

### Restricted cubic spline analyses

**Fig. 3:**
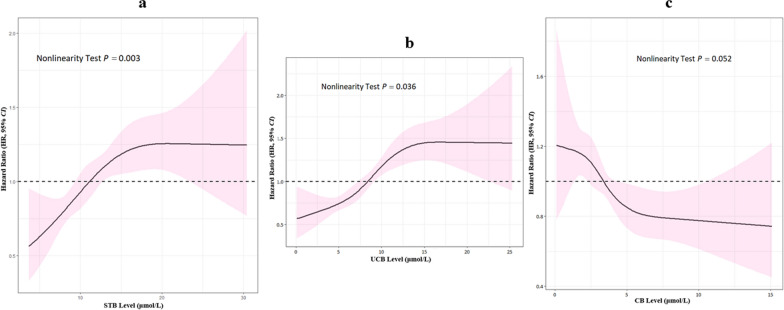
The non-linear dose–response relationship between bilirubin and hypertension. Restricted cubic spine with 4 knots was performed to determine the bilirubin (STB, UCB, and CB)-hypertension dose–response relationship. The solid blue line represents the dose–response curve with multivariable Cox model, and the pink shading indicates the 95% confidence interval. STB, UCB, and CB exhibited bidirectional effects to hypertensive risk with the threshold concentration of 12.17 μmol/L, 8.59 μmol/L, and 3.47 μmol/L, respectively

The RCS analyses have been performed to avoid the potential effects of inappropriate linearity and to check the relationship of the precise dose–response associations between predictor and response variables. As shown in Fig. 3, the *P* value of nonlinearity test (*P* = 0.003) confirmed the non-linear association between STB and hypertension. Beyond this, STB exhibited bidirectional physiological effects in different vivo concentration range with the cut-off of 12.17 μmol/L. STB levels below the cut-off point of 12.17 μmol/L were considered to show protective effects, while the opposite effects presented beyond the cut-off. The similar situation arose between hypertension incident risks and UCB in indicated the bidirectional effects of UCB with the cut-off of 8.59 μmol/L. Nevertheless, serum CB concentrations presented the unstable correlation with the hypertensive hazards, which was in contrast to STB and UCB, as 3.47 μmol/L was the cut-off point (Additional file 1: Table S2).

### The association between bilirubin and hypertension stage

In addition, participants were divided into 4 hypertension stages at the end of follow-up according to the classification requirements, including normal group of 485 subjects (25.78%), pre-hypertension group of 938 subjects (49.87%), Stage 1 hypertension group of 385 subjects (20.47%), Stage 2 of 63 subjects (3.35%), and Stage 3 of 10 subject (0.53%). Due to the number of cases greater than or equal to Stage 1 hypertension was too small, we combined these cases into one ≥ Stage 1 hypertension group. Table 5 showed that variables of age (*P* < 0.001), WC (*P* < 0.001), BMI (*P* = 0.006), ALT (*P* < 0.001), AST (*P* = 0.006), ALT (*P* = 0.006), albumin (*P* = 0.017), and TG (*P* = 0.050) exhibited different distribution and increased as the condition of hypertension got worse. Interestingly, the consistently decreasing trends in STB and UCB appeared to exist along with the deterioration of hypertension (*P*_STB_ = 0.723; *P*_UCB_ = 0.782), but an increasing trend seemed to be observed in CB (*P*_CB_ = 0.053).

**Table 5:** Participants’ characteristics of the GKASC grouped by HTN stage

Variables	Overall	Normal	Pre-HTN	≥ Stage 1 HTN	*P* value
	N = 1881	n = 485 (25.78%)	n = 938 (49.87%)	n = 458 (24.35%)	
Age (years)	65.00 (62.00–70.00)	65.00 (62.00–69.00)	65.00 (62.00–69.00)	66.00 (63.00–72.00)	< 0.001
Male (n, %)	823 (43.75)	200 (41.24)	423 (45.10)	200 (43.67)	0.380
WC (cm)	81.00 (75.00–88.00)	80.00 (74.00–87.00)	80.00 (75.00–87.00)	82.00 (76.00–89.00)	< 0.001
BMI (kg/m^2^)	22.23 (20.45–24.46)	22.06 (20.17–24.12)	22.23 (20.36–24.46)	22.54 (20.85–24.77)	0.006
Leukocyte (× 10^9^/L)	5.90 (5.10–7.00)	5.80 (4.80–6.92)	5.90 (5.10–7.10)	6.00 (5.30–7.00)	0.095
Platelet (× 10^9^/L)	217.00 (190.00–252.00)	217.00 (188.00–251.50)	217.00 (189.00–250.00)	220.00 (193.00–254.00)	0.409
Hemoglobin (g/L)	135.00 (126.00–144.00)	134.00 (126.00–143.00)	135.00 (127.00–144.00)	135.00 (126.00–145.00)	0.324
FPG (mmol/L)	4.99 (4.50–5.60)	5.00 (4.40–5.55)	4.93 (4.50–5.60)	4.98 (4.50–5.60)	0.617
ALT (U/L)	20.95 (16.33–27.50)	20.40 (15.70–25.45)	20.70 (16.50–27.50)	22.80 (17.10–30.20)	< 0.001
AST (U/L)	21.00 (18.00–24.20)	20.00 (18.00–23.90)	21.00 (18.00–24.00)	21.00 (18.00–26.00)	0.006
Albumin (g/L)	44.60 (42.90–46.00)	44.20 (42.75–45.50)	44.60 (42.80–46.10)	44.75 (43.20–46.30)	0.017
STB (μmol/L)	11.90 (9.25–15.20)	12.00 (9.10–15.10)	11.90 (9.28 -15.23)	11.85 (9.40–15.40)	0.723
CB (μmol/L)	3.20 (2.20–4.40)	3.00 (2.10–4.10)	3.10 (2.10–4.50)	3.30 (2.38–4.60)	0.053
UCB (μmol/L)	8.30 (5.90–11.45)	8.60 (5.95–11.60)	8.30 (5.70–11.50)	8.10 (6.10–11.13)	0.782
SCr (μmol/L)	62.80 (51.70–75.80)	62.70 (50.70–76.35)	63.10 (51.98–75.50)	62.60 (52.25–76.13)	0.853
TC (mmol/L)	5.03 (4.40–5.71)	5.06 (4.42–5.69)	5.05 (4.42–5.79)	5.00 (4.32–5.64)	0.107
TG (mmol/L)	1.04 (0.77–1.48)	1.00 (0.74–1.38)	1.05 (0.77–1.52)	1.07 (0.81–1.55)	0.050
T2DM (n, %)	176 (9.36)	39 (8.04)	93 (9.91)	44 (9.61)	
Hyperuricemia (n, %)	468 (24.88)	124 (25.57)	216 (23.05)	128 (27.95)	
Marriage Status (n, %)					0.486
Married	1649 (87.67)	432 (89.07)	818 (87.21)	399 (87.12)	
Widowed	220 (11.70)	50 (10.31)	116 (12.37)	54 (11.79)	
Unspecified	12 (0.64)	3 (0.62)	4 (0.43)	5 (0.11)	
Smoking Habits					0.958
Non-smoker	1395 (74.16)	365 (75.26)	689 (73.45)	341 (74.45)	
Former Smoker	59 (3.14)	14 (0.29)	30 (3.20)	15 (3.28)	
Current Smoker	427 (22.70)	106 (21.86)	219 (23.35)	102 (22.27)	
Drinking Habits					0.375
Non-drinker	831 (87.81)	425 (87.81)	800 (85.29)	390 (85.15)	
Current Drinker	121 (12.70)	59 (12.19)	138 (14.71)	68 (14.85)	
Exercise frequency					0.880
Low	1109 (58.96)	294 (60.62)	552 (58.85)	263 (57.42)	
Medium	171 (9.09)	41 (8.45)	88 (9.38)	42 (9.17)	
High	601 (31.95)	150 (30.93)	298 (31.77)	153 (33.41)	

Normally distributed variables with even variance were presented as mean ± SD, skewed variables as median (interquartile range), and categorical variables as n (%)

Continuous variables were compared using Student’s t-test or Mann–Whitney U test depending on distribution. Pearson’s χ^2^ tests were used to compare categorical values

HTN, hypertension; WC, waist circumference; BMI, body mass index; FPG, fasting plasm blood; ALT, alanine aminotransferase; AST, aspartate aminotransferase; STB, serum total bilirubin; CB, conjugated bilirubin; UCB, unconjugated bilirubin; SCr, serum creatinine; TC, total cholesterol; TG, triglyceride; T2DM, type 2 diabetes mellitus

### Model predictive abilities

**Fig. 4:**
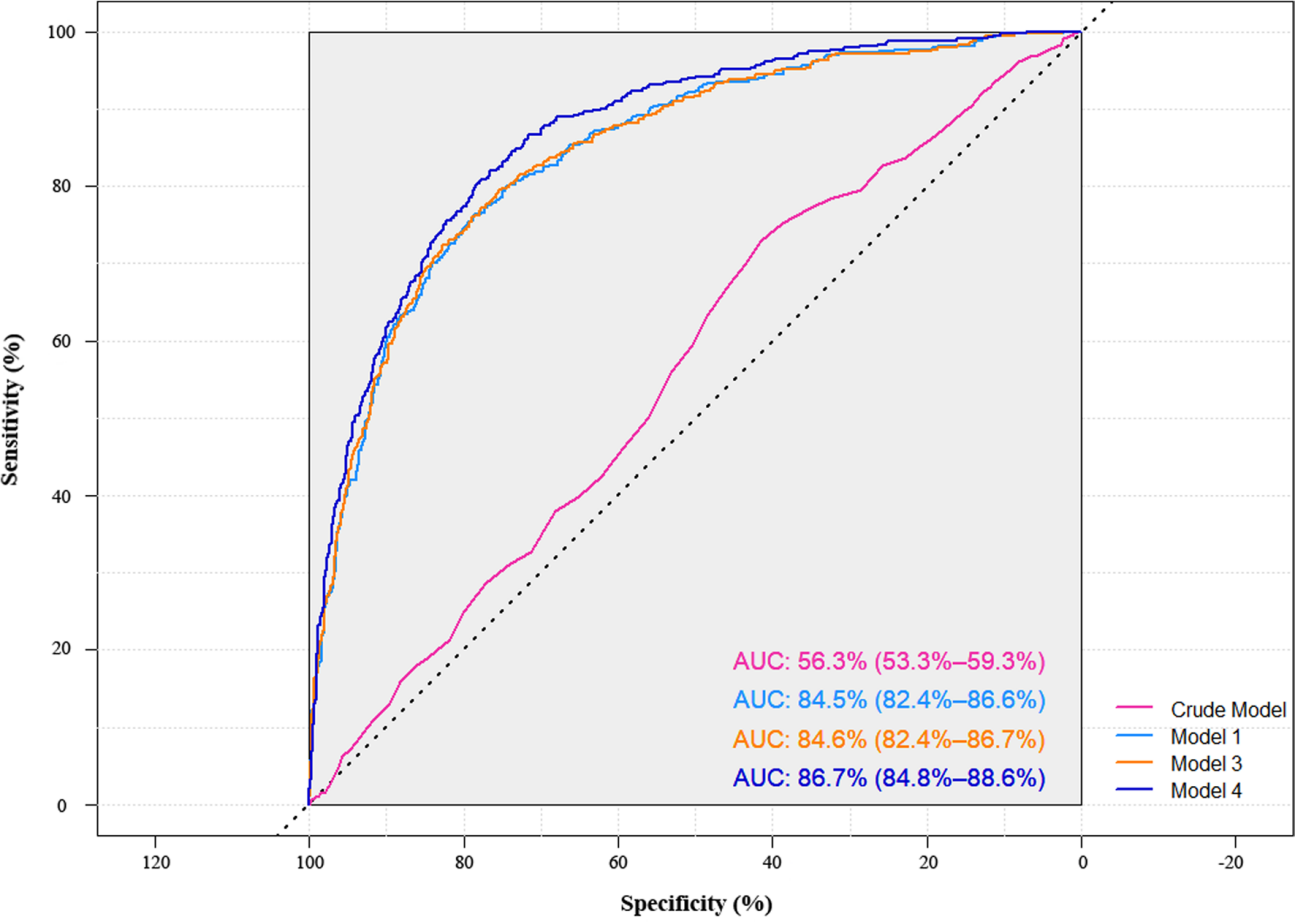
ROC curves and AUC of models. Crude model: Used STB as the single variable and unadjusted other baseline variables; Multivariable model 1: Adjusted for age, gender, BMI, and WC on the basis on crude model; Multivariable model 2: Further adjusted for SBP, DBP, and DM at baseline on the basis on model 1; Multivariable model 3: Further adjusted for FPG_*log10*_, AST, and SCr_*SQRT*_ on the basis on model 2; Multivariable model 4: Further adjusted for smoking status on the basis on model 3. The ROC curve of Model 2 had little enhancement of predictive power with the supplement of baseline SBP, DBP, and DM of on the basis of model 1. Thus, the ROC curve of Model 2 is not shown in the figure. ROC, receiver operating characteristic curve; AUC, area under curve; CB, conjugated bilirubin; BMI, body mass index; WC, waist circumference; SBP, systolic blood pressure; DBP, diastolic blood pressure; DM, diabetes mellitus; AST, aspartate aminotransferase; FPG, fasting plasma glucose; SCr, serum creatinine

Due to STB represented the co-regulating effects of CB and UCB, we constructed hypertension predicting models based on STB and evaluated their predictive powers among the ageing population by generating ROC curves and calculating AUC (Fig. 4). The discriminative performance showed a significant improvement compared with the crude model when only baseline STB was included (AUC_*Crude*_ 0.56, 95% *CI* 0.53–0.59; AUC_*Model1*_ 0.85, 95% *CI* 0.82–0.87; AUC_*Model2*_ 0.85, 95% *CI* 0.83–0.88; AUC_*Model3*_ 0.85, 95% *CI* 0.82–0.87; AUC_*Model4*_ 0.87, 95% *CI* 0.85–0.89). Both ANOVA and AIC were used to access the goodness of the above fits, which suggested the addition of baseline SBP and DBP of model 2 had little enhancement of predictive power compared with model 1 (*P*_ANOVA_ = 0.228). Regarding of the balance of cost and predicative performance, STB at baseline combined with age, BMI, and WC showed a good practical potential to discriminate the high-risk of hypertension.

**Fig. 5:**
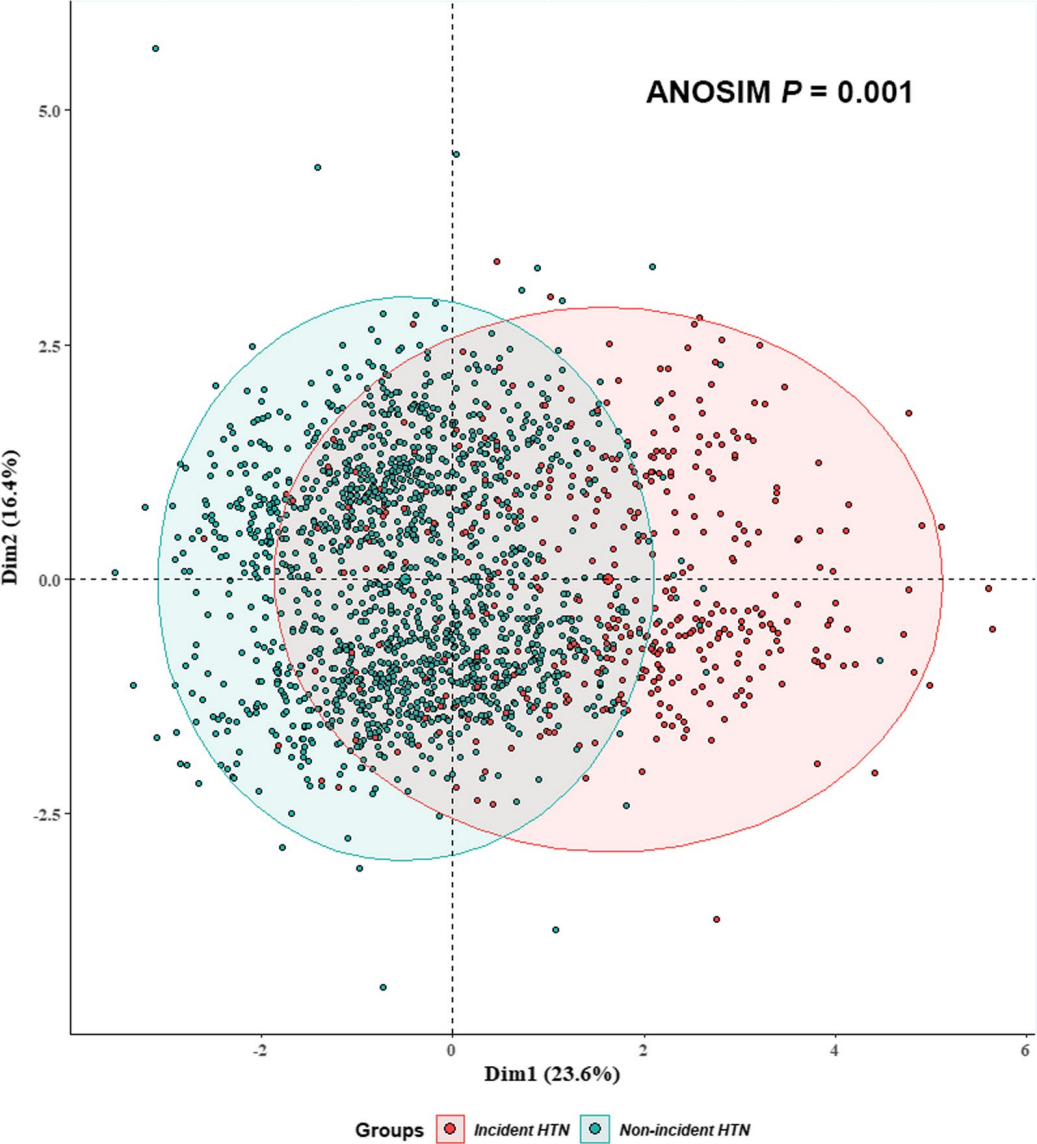
Clustering analysis based on MFA algorithm. Red dots represent participants diagnosed with hypertension at the end of follow-up, while green dots are non-incident ones. Participants with high- and low-risk hypertension were clustered into two distinct clusters with STB at baseline combined with age, BMI, and WC. Ellipses represent the 95% *CI*. BMI, body mass index; WC, waist circumference; MFA, multiple factor analysis

We conducted clustering analysis based on MFA algorithm to further examine discernibility power of Model 1, and the results supported the conclusions generated by ROC curves. Specifically, by applying MFA, we identified the first and second principal component (Dim1; Dim2) that explained most of the variance in our data (23.6% and 16.4%) as shown in Fig. 5. Research subjects with high- and low-risk hypertension could be well clustered into 2 distinct clusters with STB at baseline combined with age, BMI, and WC.

## Discussion

Hypertension represents a primary risk factor of cardiovascular and cerebrovascular diseases linked with endothelial dysfunction, which is the leading cause of mortality in the world. The population ageing, rapid urbanization, as well as the changes of environment and lifestyle count great significance for hypertension preventing and controlling [35–37]. In addition, more attention should be paid to the molecular risk factors, which may indicate the pathology and strengthen prevention of hypertension. Bilirubin could be served as one of the molecular factors that has been associated with hypertension in few studies, however, the previous conclusions are still controversial.

In our present work, we analyzed the relationship between bilirubin concentration and hypertension risk according to the data obtained from the GACS. The bidirectional effects of the different bilirubin species were observed along their concentration changes. The lower levels of STB and UCB showed the protective effects towards hypertension, while the opposite effects were observed at the higher levels. The inverse associations have been observed between serum CB and hypertension. The validity of our research is bolstered by previous research that has reached the similar conclusions. *Yasuko Takeda* et al. selected 37 patients with pulmonary arterial hypertension (PAH) as the research objects and manifested that elevated serum bilirubin is a risk factor for death in patients with PAH [18]. Beyond that, according to the research of 37,544 newborns (31,819 term and 5725 preterm births) from the U.S. Collaborative Perinatal Project conducted from 1959 to 1965, Huan Yu et al. proposed that neonatal serum bilirubin levels at 48 h after birth were positively associated with childhood blood pressure/hypertension in the preterm infants at 7 years [19]. However, other experts offered diverse perspectives that high serum bilirubin may decrease hypertensive risk. For example, Lina Wang et al. analyzed data from the National Health and Nutrition Examination Surveys (NHANES) 1999–2012 (N = 31,069) and demonstrated that SBP decreased progressively up to − 2.5 mmHg (*P* < 0.001) and the prevalence of hypertension was up to 25% lower (*P* < 0.001) in those with bilirubin ≥ 1.0 mg/dL-the highest two deciles-compared with those with 0.1–0.4 mg/dL-the lowest decile. The author supposed that the fundamental mechanism was high serum bilirubin level might inactivate and inhibit the synthesis of reactive oxygen species in vascular cells to decrease the risk of hypertension [20]. Ho Jun Chin et al. also proposed that bilirubin concentration was negatively correlated with hypertension incident risk among normotensive Korean adults [21]. Such protective effects were mainly attributed to the fact that bilirubin has significant antioxidant properties, such as preventing vitamin A and polyunsaturated fatty acids from oxidation [17]. Meanwhile, bilirubin was supposed to be a potent substance of scavenging hydrogen peroxide radicals and therefore fulfills the anti-oxidative function. However, when exploring the effects of bilirubin in some ageing-related diseases, such as cardiovascular and metabolic disorders, some large-scale cross-sectional and cohort studies have suggested the potential protective effects of bilirubin [38, 39]. We would like to offer a perspective view that STB and UCB mainly exerted both harmful and preventive influence to hypertension, which have the patterns similar to hormesis effect [40]. We also observed that CB was weakly and negatively correlated with hypertension, which was partially in agreement with those of previous studies [41]. The metabolism cross-talk of STB, UCB and CB may well illustrate the role of bilirubin, which suggested that different bilirubin metabolites should be treated dialectically when assessing their risk of hypertension.

The pathological mechanisms of hypertension remain extremely complicated. The partial characteristic of hypertension is mild symptoms of inflammation, and the main mechanisms to pathogenesis involving in the up-regulation of the sympathetic nervous system and the increased renin–angiotensin–aldosterone system (RAAS)-activity [42]. CB and UCB are the two major species of bilirubin, which display unique chemical properties, and UCB constitutes larger proportion than CB. As a lipophilic molecule, UCB is able to bind to human neurons, which is enriched in phospholipids. While the excitability of sympathetic nervous system exhibited a positive relationship with the bilirubin deposited in the central nervous system, which matters in the hypertensive pathogenesis [19, 43]. In contrast, CB presented the inverse effect as UCB, which can be plausibly interpreted by its chemical properties of water-soluble and urinary ready excretion. Therefore, phase-II conjugation is important to balance serum CB and avoid UCB accumulation. The breakthrough increasing of UCB may add hypertension risk. Anyway, how the adverse action of bilirubin initiating and/or progressing hypertension remains unclear.

Because of the bidirectional effect, bilirubin appears not to be suitable as an ideal hypertension biomarker as the AUC of crude model is relatively low (AUC_*Base*_ 0.56, 95% *CI* 0.53–0.59), but this result hinted at the significance of subsequent researches. Regarding of the balance of cost and predicative performance, Model 1 showed a good practical potential to discriminate the high-hypertension risks. In addition, the 7-year hypertension incident rate in the GACS was 6.29 per 100 person-years (5.97 and 6.51 per 100 person-years in men and women, respectively), which was relatively lower in the similar researches conducted in German [44] and Korea [45]. The possible reasons might be as follows: (1) The participants in the GACS were relatively younger than those in other researches. The CARLA study, conducted among Germany general population, was comprised of 1779 subjects with a mean age of 64.9 (SD = 10.2) years for men and 63.8 (SD = 9.9) years for women at baseline. The positive association between increasing age and incidence of hypertension was in agreement with many literatures, which could be attributed to lower-level physical activity, differences in dietary intake, the age-dependent hardening of the vascular system and worsening of kidney function [46, 47]; (2) The participants in the GACS were shown to be more “slim” than other cohorts. The male and female participants showed the mean BMI values of 22.13 (SD = 0.10) and 22.88 (SD = 0.10) in the GACS, which were relatively lower than Germany and Korean population involved. BMI is a well-recognized risk factor for hypertension [48], and in a meta-analysis, the mean SBP and DBP reductions associated with an average weight loss of 5.1 kg were 4.4 and 3.6 mmHg, respectively [49]; (3) Another possible reason might be attributed to the annual routine health examination of the subjects, which may be the relatively healthy part of the population have been involved.

In our study, the prevalence of T2DM and hyperuricemia were not significantly different between genders. He et al. used real-world data to estimate the changing tendencies in the prevalence of T2DM in Xiamen City from 2014 to 2019. Interestingly, the overall prevalence among the male and female adults were 4.18% and 5.52% in Xiamen, and T2DM prevalence exhibited an increasing trend with advancing age regardless of gender [50]. In our work, female participants were younger than males, which could be one possible reason to explain the prevalence of T2DM were not significantly different between genders. The discrepancy of gender-specific hyperuricemia prevalence is also existed, which might be attributed to the lifestyles, dietary habits, regional economic level, and individual living standards [51, 52]. Besides, women in post-menopausal could lead to estrogen deficiency. Estrogens may promote renal clearance of serum urate and its deficiency can result in changes in the endocrine system and increase of metabolic diseases [53]. However, the underlying mechanisms that lead to the gender-specific prevalence of hyperuricemia requires further exploration.

In our risk discrimination analysis, the predictive performance of model exhibited a stepwise increase with the processive inclusion of significant associated factors and relevant biological variables, especially in Model 4. A large body of previous studies proposed smoking was a risk factor for hypertension [54–56], thus, the ability of risk discrimination in Model 4 improved drastically. However, there were very few differences of the predictive performance between Model 1 and Model 3, which hinted variables of age, gender, BMI, and WC could be considered as the main parameters in the risk discrimination model. Moreover, as shown in Crude Model, the AUC was relatively low (AUC_Base_ 0.56, 95% CI 0.53–0.59), which suggested STB appears not to be suitable as an ideal hypertension biomarker because of the bidirectional effect. Regarding of the balance of cost and predicative performance, Model 1 showed a good practical potential to screen the subjects with high-hypertension risks.

The current analyses had several strength and limitations. The first strength was that our research was based on a large prospective study, the GACS with high-quality design, and the tracing of diseases made it possible to recognize potential protective and harmful factors as well as their modes of action on ARDs. The data of physiology, lifestyle factors, socioeconomics, and biological samples during the long-term follow-up increased our confidence to infer the underlying mechanisms of certain questions raised from the observation. And the treatment regimens based on bilirubin in clinical practice may be available when it is verified by the future studies. As for limitations, one of the limitations was hypertensive state is unstable, which can be affected by environmental and psychological causes, and the case might inevitably include the false positive confirmation. The standard definition of hypertension clinical diagnosis was based on the three BP measurements in at least two different occasions [57]. The BP readings of our subjects were measured only in the same hospital. It is also difficult to require subjects to use 24 h blood pressure monitoring equipment during the follow-up. The second limitation was hypertension subtypes (primary and secondary hypertension) were not well discriminated because of technical and cost limits during follow-up, which might introduce bias into further analysis. At last, the state of hyperuricemia in our research was relied on anamnesis and medical records of participants, rather than dynamic monitoring data, which might pose a potential risk of bias towards our conclusions.

All in all, this study provides the valuable clues of the associations between bilirubin and hypertension, even though the underlying mechanisms remain ambiguous to date. Advancing theories and methodologies is urgently called for to uncover the bilirubin-hypertension association from the more refined perspective [58].

## Conclusions

Our findings suggested STB and UCB exhibited consistently hazardous effects to the incident risk of hypertension higher levels after multivariable adjustments, while an inverse effect could be observed in conjugated bilirubin. And current findings did not identify an association between bilirubin level and hypertension severity.

## Supplementary Information

**Additional file 1.** The supplementary files of this article

## Data Availability

The datasets used and/or analyzed during the current study are available from the corresponding author on reasonable request.

## Abbreviations

ALT: Alanine transaminase
ANOVA: Analysis of variance
ARDs: Ageing-related diseases
AST: Aspartate aminotransferase
BMI: Body mass index
BP: Blood pressure
CB: Conjugated bilirubin
*CI*: Confidence intervals
DBP: Diastolic blood pressure
T2DM: Type 2 diabetes mellitus
FPG: Fasting plasma glucose
GACS: Guankou Ageing Cohort Study
MFA: Multiple factor analysis
MICE: Multiple imputation by chained equations
HR: Hazard rations
SBP: Systolic blood pressure
SCr: Serum creatinine
SQRT: Square root
STB: Serum total bilirubin
UCB: Unconjugated bilirubin
WC: Waist circumference
