# Supplementary material for: The association between bilirubin and hypertension among a Chinese ageing cohort: a prospective follow-up study
Source: J Transl Med. 2022 Mar 4;20:108. doi: 10.1186/s12967-022-03309-7 (PMC8895624; doi:10.1186/s12967-022-03309-7)
Supplement: Supplementary file 1 — Additional file 1. The supplementary files of this article [file 12967_2022_3309_MOESM1_ESM.docx]

| **Table S1. Characteristic survey of the eligible participants at recruitment** | | | | | |
| --- | --- | --- | --- | --- | --- |
| Variables | | Overall | Baseline HTN (-) | Baseline HTN (+) | ***P*** value |
|  |  | N=5755 | n=1881 (32.7%) | n=3874 (67.3%) |  |
| Age (years) | | 62.00 (60.00-68.00) | 61.00 (59.00-66.00) | 63.00 (60.00-69.00) | <0.001 |
| Male (n, %) | | 2442 (42.43) | 823 (43.75) | 1619 (41.79) | 0.158 |
| WC (cm) | | 83.92 ± 18.44 | 81.19 ± 9.30 | 85.25 ± 21.40 | <0.001 |
| BMI (kg/m^2^) | | 23.50 ± 3.39 | 22.56 ± 3.18 | 23.95 ± 3.39 | <0.001 |
| Leukocyte (×10^9^/L) | | 6.00 (5.24-7.10) | 5.83 (5.10-7.00) | 6.10 (5.30-7.20) | <0.001 |
| Platelet (×10^9^/L) | | 218.00 (190.00-252.00) | 217.00 (190.00-252.00) | 218.00 (191.00-252.00) | 0.933 |
| Hemoglobin (g/L) | | 136.00 (127.00-145.00) | 135.00 (126.00-144.00) | 136.0 (127.00-145.00) | <0.001 |
| FPG (mmol/L) | | 5.10 (4.50-5.80) | 4.99 (4.50-5.60) | 5.10 (4.59-5.90) | <0.001 |
| ALT (U/L) | | 22.30 (17.10-29.70) | 20.90 (16.30-27.50) | 22.95 (17.50-30.70) | <0.001 |
| AST (U/L) | | 21.00 (18.00-25.00) | 21.00 (18.00-24.10) | 21.00 (18.00-25.80) | <0.001 |
| Albumin (g/L) | | 44.90 (43.10-46.50) | 44.50 (42.90-46.00) | 45.00 (43.30-46.70) | <0.001 |
| STB (μmol/L) | | 11.90 (9.30-15.20) | 11.90 (9.25-15.20) | 11.90 (9.30-15.20) | 0.841 |
| CB (μmol/L) | | 3.20 (2.20-4.50) | 3.20 (2.20-4.50) | 3.20 (2.20-4.50) | 0.192 |
| UCB (μmol/L) | | 8.30 (5.80-11.40) | 8.30 (5.90-11.45) | 8.30 (5.80-11.40) | 0.942 |
| SCr (μmol/L) | | 62.50 (51.30-76.30) | 62.80 (51.70-75.80) | 62.20 (51.10-76.50) | 0.790 |
| TC (mmol/L) | | 5.06 (4.39-5.77) | 5.03 (4.40-5.71) | 5.07 (4.39-5.81) | 0.175 |
| TG (mmol/L) | | 1.18 (0.83-1.73) | 1.04 (0.77-1.48) | 1.26 (0.88-1.85) | <0.001 |
| Marriage Status (n, %) | |  |  |  | <0.001 |
| Married | | 4840 (84.10) | 1638 (87.08) | 3202 (82.65) |  |
| Widowed | | 869 (15.10) | 229 (12.17) | 640 (16.52) |  |
| Unspecified | | 46 (0.80) | 14 (0.74) | 32 (0.82) |  |
| Smoking Status | |  |  |  | <0.001 |
| Non-smoker | | 4220 (73.60) | 1331 (71.00) | 2889 (74.80) |  |
| Former Smoker | | 206 (3.60) | 53 (2.80) | 153 (4.00) |  |
| Current Smoker | | 1311 (22.90) | 491 (26.20) | 820 (21.20) |  |
| Drinking Status | |  |  |  | 0.875 |
| Non-drinker | | 4762 (83.30) | 1560 (83.40) | 3202 (83.20) |  |
| Current Drinker | | 957 (16.70) | 311 (16.60) | 646 (16.80) |  |
| Exercise Frequency | |  |  |  | <0.001 |
| Low | | 3194 (55.50) | 1102 (58.60) | 2092 (54.00) |  |
| Medium | | 960 (16.70) | 322 (17.10) | 638 (16.50) |  |
| High | | 1601 (27.80) | 457 (24.30) | 1144 (29.50) |  |

Normally distributed variables with even variance were presented as mean ± SD, skewed variables as median (lower quartile to upper quartile), and categorical variables as n (%).

Continuous variables were compared by using Student’s t-test or Mann-Whitney U test depending on the distribution. Pearson’s χ^2^ test was used to compare categorical values.

Abbreviations: HTN, hypertension; WC, waist circumference; BMI, body mass index; FPG, fasting plasm glucose; ALT, alanine aminotransferase; AST, aspartate aminotransferase; STB, serum total bilirubin; CB, conjugated bilirubin; UCB, unconjugated bilirubin; SCr, serum creatinine; TC, total cholesterol; TG, triglyceride.

**Table S2. Prospective analysis of associations between CB levels and hypertension incidence in the GACS**

|  | CB quartiles | | | | |  |
| --- | --- | --- | --- | --- | --- | --- |
|  | 1  <2.2 μmol/L | 2  2.2~3.1 μmol/L | 3  3.2~4.4 μmol/L | 4  >4.4 μmol/L | ***P*** for trend |  |
| Crude Model | Reference | 1.00 (0.73-1.35) | 0.85 (0.63-1.15) | 0.74 (0.55-1.01) | 0.106 |  |
| Model 1 | Reference | 0.74 (0.54-1.01) | 0.67 (0.49-0.91) | 0.60 (0.44-0.83) | 0.015 |  |
| Model 2 | Reference | 0.75 (0.55-1.02) | 0.65 (0.48-0.89) | 0.60 (0.44-0.82) | 0.011 |  |
| Model 3 | Reference | 0.85 (0.62-1.16) | 0.79 (0.58-1.09) | 0.70 (0.51-0.96) | 0.167 |  |
| Model 4 | Reference | 0.84 (0.61-1.15) | 0.81 (0.59-1.12) | 0.65 (0.47-0.90) | 0.054 |  |

Multivariable-adjusted Cox regression models were used to assess the hypertension incidence risk by CB quartiles.

Multivariable model 1: Adjusted for age, gender, BMI, and WC.

Multivariable model 2: Further adjusted for SBP, DBP, and DM at baseline.

Multivariable model 3: Further adjusted for FPG*_log10_*, AST, and SCr*_SQRT_*.

Multivariable model 4: was further adjusted for smoking status.

Abbreviations: CB, conjugated bilirubin; BMI, body mass index; WC, waist circumference; SBP, systolic blood pressure; DBP, diastolic blood pressure; DM, diabetes mellitus; AST, aspartate aminotransferase; FPG, fasting plasma glucose; SCr, serum creatinine.

**Table S3. Prospective analysis of associations between UCB levels and hypertension incidence in the GACS**

|  | UCB quartiles | | | | |  |
| --- | --- | --- | --- | --- | --- | --- |
|  | 1  <5.9 μmol/L | 2  5.9~8.2 μmol/L | 3  8.3~11.4μmol/L | 4  >11.4 μmol/L | ***P*** for trend |  |
| Crude Model | Reference | 1.15 (0.87-1.51) | 1.54 (1.18-2.00) | 1.84 (1.40-2.41) | <0.001 |  |
| Model 1 | Reference | 1.08 (0.82-1.42) | 1.63 (1.25-2.12) | 2.26 (1.72-2.98) | <0.001 |  |
| Model 2 | Reference | 1.11 (0.84-1.46) | 1.63 (1.25-2.13) | 2.18 (1.65-2.88) | <0.001 |  |
| Model 3 | Reference | 1.18 (0.89-1.56) | 1.70 (1.30-2.22) | 2.07 (1.57-2.74) | <0.001 |  |
| Model 4 | Reference | 1.30 (0.98-1.72) | 1.67 (1.28-2.19) | 2.03 (1.54-2.69) | <0.001 |  |

Multivariable-adjusted Cox regression models were used to assess the hypertension incidence risk by UCB quartiles.

Multivariable model 1: Adjusted for age, gender, BMI, and WC.

Multivariable model 2: Further adjusted for SBP, DBP, and DM at baseline.

Multivariable model 3: Further adjusted for FPG*_log10_*, AST, and SCr*_SQRT_*.

Multivariable model 4: was further adjusted for smoking status.

Abbreviations: UCB, unconjugated bilirubin; BMI, body mass index; WC, waist circumference; SBP, systolic blood pressure; DBP, diastolic blood pressure; DM, diabetes mellitus; AST, aspartate aminotransferase; FPG, fasting plasma glucose; SCr, serum creatinine.

**Table S4. P values of pairwise comparisons between quartiles**

|  | Quartile 1 | Quartile 2 | Quartile 3 | Quartile 4 |
| --- | --- | --- | --- | --- |
| Quartile 1 | - | 0.382 | 0.013 | 0.003 |
| Quartile 2 | 0.382 | - | 0.097 | 0.028 |
| Quartile 3 | 0.013 | 0.097 | - | 0.596 |
| Quartile 4 | 0.003 | 0.028 | 0.596 | - |
